# Microstructure and Selected Properties of Advanced Biomedical n-HA/ZnS/Sulfonated PEEK Coatings Fabricated on Zirconium Alloy by Duplex Treatment

**DOI:** 10.3390/ijms23063244

**Published:** 2022-03-17

**Authors:** Filip Kuśmierczyk, Aleksandra Fiołek, Alicja Łukaszczyk, Agnieszka Kopia, Maciej Sitarz, Sławomir Zimowski, Łukasz Cieniek, Tomasz Moskalewicz

**Affiliations:** 1 Faculty of Metals Engineering and Industrial Computer Science, AGH University of Science and Technology, al. Mickiewicza 30, 30-059 Kraków, Poland; kruka@agh.edu.pl (A.F.); kopia@agh.edu.pl (A.K.); lukasz.cieniek@agh.edu.pl (Ł.C.); 2 Faculty of Foundry Engineering, AGH University of Science and Technology, al. Mickiewicza 30, 30-059 Kraków, Poland; alicjal@agh.edu.pl; 3 Faculty of Materials Science and Ceramics, AGH University of Science and Technology, al. Mickiewicza 30, 30-059 Kraków, Poland; msitarz@agh.edu.pl; 4 Faculty of Mechanical Engineering and Robotics, AGH University of Science and Technology, al. Mickiewicza 30, 30-059 Kraków, Poland; zimowski@agh.edu.pl

**Keywords:** zirconium alloy, bioactive coating, sulfonated PEEK, electrophoretic deposition, adhesion strength, corrosion resistance

## Abstract

In this work, sulfonated polyetheretherketone (S-PEEK)-based coatings, nanocrystalline ZnS and hydroxyapatite (n-HA) particles were developed on Zr-2.5Nb zirconium alloy substrates by electrophoretic deposition (EPD) combined with subsequent heat treatment. The properties of suspensions and deposition kinetics were studied. Cationic chitosan polyelectrolyte ensured the stabilization of the suspension and allowed for the co-deposition of all coating components on the cathode. The heating of the coated samples at a temperature of 450 °C and slow cooling resulted in sulfonation of the PEEK and the formation of dense coatings. The coatings were characterized by high roughness, hardness, modulus of elasticity and adhesion strength. The coatings revealed mild hydrophilicity, improved the electrochemical corrosion resistance of the alloy and induced the formation of hydroxyapatite with a cauliflower-like morphology on its surface during the Kokubo test. This work explored the great development potential of advanced sulfonated PEEK-based coatings, incorporating antibacterial and bioactive components by EPD combined with heat treatment to stimulate the surface properties of zirconium alloy for prospective dental and orthopedic applications. The antibacterial and osteoconductive properties of the obtained coatings should be further investigated.

## 1. Introduction

Zirconium alloys are among the most suitable metallic biomaterials generally exploited as bone implants, i.e., dental and orthopedic replacements [1,2,3]. Their importance in biomedical applications results from their outstanding properties, such as high electrochemical corrosion resistance, biocompatibility, elasticity modulus of around 90 GPa and density of 6.5 g/cm^3^ in the range between titanium alloys and stainless steels [4,5]. The Zr-2.5Nb alloy is one of the best known among biomedical zirconium alloys. It exhibits higher strength, hardness, wear resistance and electrochemical corrosion resistance than that of pure Zr, as well as good in vitro cytocompatibility and emphatically lower magnetic susceptibility than the widely-used cobalt-based alloys and Ti-6Al-4V alloy [6]. Although Zr-2.5Nb biocompatibility is excellent, it has poor osteointegration with bone cells [5,7]. One of the most promising ways of improving its biological properties is the development of bioactive coatings.

The use of polymers as coating materials is beneficial in comparison to metals, because of the reduction in weight and higher electrochemical corrosion resistance. In recent years, polyetheretherketone (PEEK) has been widely studied as a suitable coating material [8,9,10,11]. PEEK is a semi-crystalline, high-performance polymer. It is characterized by its outstanding chemical and thermal stability, elastic modulus (3–4 MPa) that is similar to human cancellous bone, good tribological properties, low density (1.32 g/cm^3^) and stiffness [10,12,13]. Therefore, it is often used as a replacement for metals in high-end applications, including medical equipment and implants. It is insolvable in water and resistant to a wide range of bases, hydrocarbons, organic solvents and acids [14,15,16,17,18]. PEEK is recognized as having one of the highest levels of chemical stability, mechanical strength and biocompatibility among other biocompatible synthetic polymers. It was found [9,18,19,20,21] that the coatings with a PEEK matrix exhibited superior adhesion to metallic substrates, which is crucial for every application. Although it has outstanding properties, PEEK is bioinert [12,18,22,23]. For this reason, various approaches have been used to strengthen these properties, e.g., by introducing bioactive and antibacterial agents into PEEK and sulfonation by chemical treatment with the use of sulfuric acid. In the present work, we propose the use of those two approaches simultaneously by (i) introducing hydroxyapatite (HA) and ZnS nanoparticles into PEEK and (ii) subsequent novel thermal sulfonation combined with the heat treatment of coated zirconium alloy.

HA is one of the most effective and versatile bioactive materials. It is calcium phosphate with the molecular formula Ca_10_(PO_4_)_6_(OH)_2_. This form of calcium phosphate is the least soluble and the most stable of all [24]. Moreover, this bioceramic is an accepted object of research due to its similarity to an inorganic fraction of bone. The presence of hydroxyapatite induces bone formation, which is necessary for implant osteointegration providing excessive fixation to the human bone [18]. Despite its beneficial properties, HA is brittle and has limited resistance to bacterial infections, which are among the most important implant failure factors. The most effective way to overcome this drawback is via the application of an antibacterial agent in coatings. The most important antiseptic agents competing with harmful antibiotics involve metals, their ions and compounds [25,26,27,28]. A prospective coating component with an antibacterial function is zinc sulfide, which is newly studied in this regard [29,30]. The ZnS nanoparticle is a potential inorganic antibacterial agent due to its high stability, antimicrobial activity and non-toxicity [29]. Monodisperse zinc sulfide nanospheres exhibited high antibacterial activity against, for example, the strain of *E. coli* bacteria [30,31]. In addition, sulfur is a biogenic element that can increase bioactivity and antimicrobial properties. The sulfur content of this compound may also be a potential source of this element that is required for the thermal sulfonation process of PEEK. Recent studies [32,33] confirmed that sulfonated polyetheretherketone (S-PEEK) exhibits antibacterial properties. Moreover, PEEK sulfonated to an appropriate degree can exceed proliferation and osteogenesis [32]. The most widely used method of PEEK sulfonation is its immersion in concentrated sulfuric acid [32,33,34]. Despite its versatility, this technique has been reported to have a negative effect on living cells and their DNA, due to sulfur dioxide (SO_2_) originating from the use of acid and the production of oxygen free radicals caused by sulfur compounds of low valence [35,36]. In addition, such treatment is not acceptable for coatings due to their easy detachment from metallic substrates. In our study, we propose novel thermal sulfonation combined with heat treatment of the coating, which allows PEEK to be efficiently sulfonated without sulfuric acid. As far as we are aware, no studies have been reported on the development of S-PEEK coatings containing dual antibacterial and bioactive components, which can conceivably improve the osteointegration effect. The application of this coating type is potentially beneficial as it is superior to coated metallic bone implants.

One of the most advantageous surface-engineering methods of developing polymer-based coatings with ceramic particles is electrophoretic deposition (EPD) [9,11,14,18,20,21,22,37,38,39,40]. The process consists of the migration of charged particles immersed in the suspension in the presence of an electric field toward an oppositely charged electrode. Then, particles deposit on the electrode and form the coating. As for the PEEK coatings, subsequent heat treatment is necessary for their densification. The literature regarding PEEK-based coatings developed by the EPD procedure concerns coatings with osteogenic factors, such as sol-gel glass [17], HA [11,18] as well as bioglass [9,14,21,22,38] and coatings with antibacterial agents involving silver [21] and MoS_2_ [20,41]. Bastan et al. [18] concluded that the addition of HA stimulates in vitro bioactivity in HA/PEEK coatings. Advanced PEEK-based composite coatings have been developed using EPD with both osteointegration and antiseptic ingredients. Ur Rehman et al. [15] fabricated PEEK coatings with the addition of lawsone, chitosan and bioglass, while Virk et al. [16] introduced curcumin, bioactive glass and hexagonal boron nitride into the PEEK matrix. In both studies, the coatings showed antibacterial activity, and the formation of an apatite-like layer during the bioactivity test was achieved. However, these coatings were fabricated as multilayers, which usually requires multiple operations during EPD. According to our knowledge, there is a limited number of studies on obtaining multicomponent PEEK-based complexes with bioactive and antibacterial factors simultaneously in one operation [11,20,21]. Seuss et al. [21] reported that electrophoretically deposited PEEK coatings with the addition of Ag particles and bioglass particles provided antibacterial activity. Abdulakareem et al. [11] proved the antiseptic capability of chitosan in HA and chitosan coatings with the addition of PEEK. However, in both of the above-mentioned references, bioactivity studies were not provided.

The aim of this study was to develop a duplex route based on electrophoretic co-deposition and subsequent heat treatment for obtaining multicomponent n-HA/ZnS/S-PEEK coatings on the Zr-2.5Nb alloy, which was used as a substrate material for EPD for the first time. The EPD kinetics were systematically studied and deposition conditions were optimized. The coating surface topography, microstructure and selected properties, including electrochemical corrosion resistance, micromechanical properties, adhesion strength and scratch resistance of the coatings, were investigated.

## 2. Materials and Methods

### 2.1. Materials

A Zr-2.5Nb zirconium alloy was used as a substrate for coating deposition. It was supplied by Luoyang Dingding Tungsten and Molybdenum Materials Co., Ltd. (Luoyang, China). A rod with a diameter of 20 mm was cut into discs with a thickness of 3 mm. The discs were ground with sandpaper with a final grade of 600. PEEK (VICOTE 704) powder with a particle size of up to 10 µm was provided by Victrex Europa GmbH, Hofheim am Taunus, Germany. ZnS nanoparticles with a size up to 100 nm were supplied by Nanoshel UK Ltd. (Congleton, U.K.). The n-HA nanoparticles (mean size up to 10 nm, according to the supplier) were produced by the Institute of High Pressure Physics of the Polish Academy of Sciences (Warsaw).

### 2.2. Coating Deposition and Duplex Treatment Process

**Figure 1:**
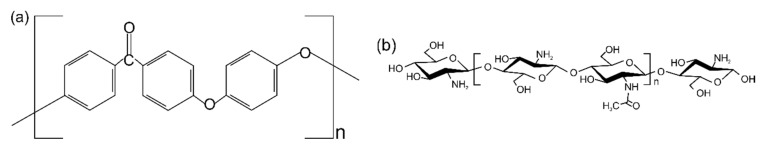
Chemical structures of PEEK (**a**) based on [42] and chitosan (**b**) based on [43].

Suspensions containing 30 g/L of PEEK 704, 0.4 g/L of ZnS and 1 g/L of n-HA, were used for coating deposition. A mixture of ethanol (with 99.8% purity) and chitosan polyelectrolyte (CHp), with a content of 95 and 5 vol %, respectively, was used as the liquid phase in suspensions. CHp was prepared by dissolving 0.5 g/L of chitosan powder and 1% acetic acid in distilled water by mixing at 600 rpm for 3 days at 23 °C with the use of a magnetic stirrer (IKA RO 5, Burladingen, Germany). The chemical structures of the PEEK and chitosan are presented in Figure 1. After the addition of ZnS and PEEK into the ethanol-CHp solution, it was magnetically stirred for 10 min and ultrasonically dispersed for 20 min to separate the agglomerates of particles. After the addition of n-HA powder, the suspension was further mixed and dispersed for 10 min. Zeta potential (ZDP) of the suspensions with respect of their pH values in the range of 3–12 was determined using a Zetasizer Nano ZS 90 that was equipped with an MPT-2 multi-purpose titrator of Malvern Instruments Ltd. (Malvern, U.K.). The electrophoretic light scattering technique based on the Doppler effect was used for investigations. The changes in the pH of mixtures were made by adding hydrochloric acid (HCl) or sodium hydroxide (NaOH).

The EPD process was executed in a two-electrode system, using an EX752M Multi-mode PSU power supply (AIM TTI, Huntingdon, UK) used as a direct current (DC) source. Zirconium alloy was the working electrode, and the counter electrode was an austenitic stainless steel plate. The constant current voltage range from 10 V to 150 V (with a change of 20 V) and a stable deposition time of 30 s were employed. A Tektronix DMM 4040 multimeter (TEKTRONIX, Bracknell, UK) was utilized to measure the current density during the process. The deposition yield and rate of particles were studied during deposition at a constant voltage of 90 V and different times of 10 s, 20 s and 30 s. The samples were weighed using an Ohaus Europe GmbH analytical scale (Nänikon, Switzerland).

Heat treatment of the samples was carried out in a Czylok MRT-20 (Czylok, Jastrzębie-Zdrój, Poland) laboratory furnace. It consisted of heating at 450 °C for 30 min (heating rate of 15 °C/min) and cooling (rate of 2 °C/min).

### 2.3. Characterization: Microstructure and Surface Topography

Microstructural investigations of the substrate, coating components and coatings were carried out using a light microscope (LM) from OPTA-TECH SK (Warsaw, Poland), FEI Nova NanoSEM 450 (FEI, Eindhoven, The Netherlands) scanning electron microscope (SEM) and JEOL JEM-2010 ARP (JEOL, Tokyo, Japan) transmission electron microscope (TEM). The lamella from the cross-section of the coating for TEM investigations was prepared using a focused ion beam (FIB) using an FEI QUANTA 3D 200i device (FEI, Eindhoven, The Netherlands). A phase analysis was performed by X-ray diffractometry (XRD) in the Bragg-Brentano arrangement using a Panalytical Empyrean DY1061 diffractometer (Malvern Panalytical, Almelo, The Netherlands) and by the selected area electron diffraction (SAED) in TEM. The patterns were interpreted with the use of a JEMS diffraction simulation software, Switzerland. The chemical composition of the coatings was analyzed using energy-dispersive X-ray spectroscopy (EDS). Structural studies in the middle infra-red region were carried out using a Bruker Vertex 70v vacuum spectrometer (Bruker, Billerica, MA, USA). The Harrick Scientific Seagull adapter was used to conduct measurements by means of external reflection spectroscopy. A total of 128 scans with a resolution of 4 cm^−1^ were collected.

The surface roughness of the coatings was examined using a Filmetrics Profilm3D non-contact optical profilometer (Filmertics, San Diego, CA, USA). Several images of the surface topography of the areas of 400 μm × 500 μm were obtained at various locations from the sample and analyzed with Filmetrics Profilm (Filmetrics, San Diego, CA, USA) software.

### 2.4. Characterization: Selected Properties

The wetting angle (WA) and interfacial free energy (IFE) of materials were examined with a Krüss DSA25E goniometer (Krüss, Hamburg, Germany), by applying distilled water (as the polar liquid) and diiodomethane (as the nonpolar liquid). Measurements were made 10 times using 10 drops of each liquid. The IFE was calculated using the Owens–Wendt–Rabel–Kaelble (OWRK) method.

Cross-cut adhesion tests were performed utilizing a cutting knife from Elcometer (Manchester, UK) in agreement with ASTM D3359B. A mesh was cut in the coating, then the tape was glued and torn off after 90 s. The surface of the samples after the tests was observed with the naked eye as well as using LM and SEM. After that, the coating area removed from the alloy surface was measured and the adhesion class was determined.

Micro-scratch tests were conducted using the Micro Combi Tester (MCT) from CSM Instruments (Peseux, Switzerland). A Rockwell C diamond stylus with an apex angle of 120° and a tip radius of 200 μm was used. The test parameters were as follows: load 0.01 N to 30 N (linear increasing), scratch length 5 mm and sample speed 5 mm/min. Microhardness and elastic modulus of the coatings were examined using the instrumental indentation technique according to the procedure described by Oliver and Pharr [44]. The Vickers indenter was pressed into the surface of the coatings with a load of 100 mN, with a constant loading and unloading rate of 200 mN/min. The dwell time under the maximum load was 15 s. The measurements were made at least ten times, each time in a different area of the coating.

A three-electrode cell assembly consisted of the working electrode (sample), a counter electrode (Pt) and a reference electrode (saturated calomel electrode) immersed in electrolyte of 8.6 g/L of NaCl, 0.3 g/L of KCl, and 0.25 g/L of CaCl_2_ dissolved in 1 L of water. The electrolyte with a pH value of 7.4 was deaerated and had a constant temperature of 37 °C. Open-circuit potential (OCP), Linear Sweep Voltammetry (LSV) and Electrochemical Impedance Spectroscopy (EIS) were carried out using an AUTOLAB PGSTAT128n potentiostat/galvanostat (Metrohm Autolab, Utrecht, The Netherlands). LSV was acquired using a scan rate of 1 mV/s, starting at −1.3 V to 2.2 V. For the EIS measurements, an amplitude of the perturbation signal ΔV = 10 mV was employed and the frequency ranged from 10^5^ Hz to 10^−3^ Hz. The EIS data were fitted using AUTOLAB NOVA software by Metrohm Autolab, The Netherlands. Error minimization for fitting an equivalent circuit was performed by means of chi-squared criteria.

To evaluate the apatite formation ability, the heat-treated n-HA/ZnS/S-PEEK-coated alloy was immersed in 1.5 simulated body fluid (SBF) for three weeks in accordance with the modified Kokubo method described by Tanahashi et al. [45]. SBF was changed weekly. After the soaking period, samples were investigated with SEM (EDS) and Raman spectroscopy. Raman measurement was performed with the use of a WITec Alpha 300M+ spectrometer (WITec Ulm, Germany). A 100× objective along with a 488 nm diode laser and 600 grating were used. The laser power was selected in such a way as to avoid the degradation of the sample.

## 3. Results and Discussion

**Figure 2:**
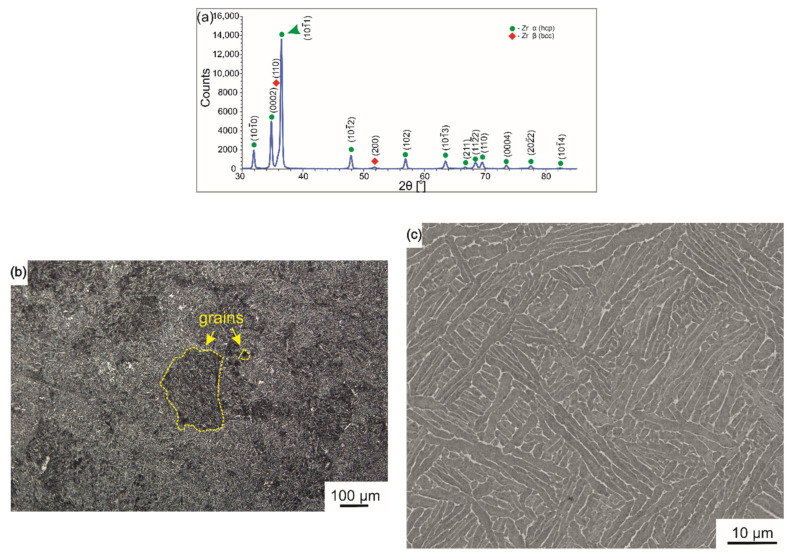
XRD pattern of Zr-2.5Nb alloy (**a**) and microstructure of the cross-section of the bar observed using LM (**b**) and SEM (**c**).

Based on the XRD phase analysis, it was detected that the Zr-2.5Nb alloy consisted principally of α phase with a hexagonal close packed (hcp) structure and sporadically of β phase with a body centered cubic (bcc) structure (Figure 2a). Based on LM observations, grains of the primary β phase had equivalent diameters in the range of 50–500 μm (Figure 2b). The SEM observation revealed that the α phase occurred in the form of numerous, elongated plates with an average width of 1.5 μm and length in the range of 0.5–30 μm (Figure 2c). The almost parallel plates formed colonies of differently oriented systems. According to the literature [46], β is an Nb-rich phase that occurs as a result of the prolonged aging of alloys quenched below the monotectoid temperature. The microstructure of the Zr-2.5Nb alloy was similar to that obtained in the same alloy by Srivastava et al. [47] after hot extrusion.

**Figure 3:**
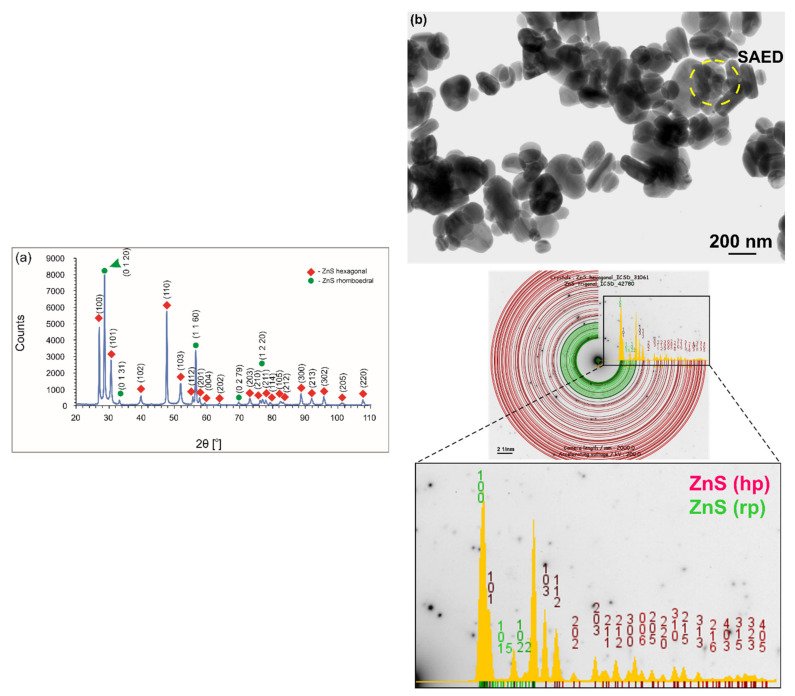
XRD pattern (**a**) and TEM micrograph (**b**) of ZnS particles. (**b**) also shows the SAED pattern obtained from the area marked by a circle and its interpretation with JEMS software.

Interestingly, PEEK 704 powder has been investigated previously and described elsewhere [17,48]. They involve amorphous particles with an irregular shape and equivalent diameter ranging from 2 to 15 μm. The ZnS powder used for coating deposition consisted of rhombohedral primitive (rp) and hexagonal primitive (hp) phases (Figure 3a). The XRD pattern displayed both intensive diffraction peaks of the rp phase of (1 1 20) and (1 1 60) and hp phase of (100) and (110) as well as minor diffraction peaks of both phases. A representative TEM micrograph of the particles is presented in Figure 3b. The particles had an equivalent diameter in the range of 40–430 nm. The following two particle shapes were observed: oblong with an average equivalent diameter of about 200 nm and globular with an equivalent diameter in the whole above-mentioned range (Figure 3b). The analysis of SAED patterns confirmed the presence of particles with both hp and rp crystallographic structures.

**Figure 4:**
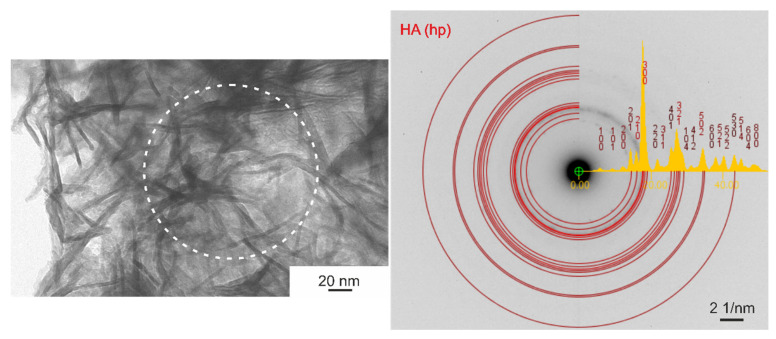
HA nano-particles and electron diffraction pattern from the area marked by the circle, TEM.

The n-HA powder contained fine longitudinal nanoparticles that exhibiting a needle-like shape with length in the range of 20–120 nm and width of about 5 nm (Figure 4). Electron diffraction analysis revealed the hp structure of particles. The Ca:P atomic ratio obtained from SEM-EDS microanalysis (analyzed area of 540 μm × 430 μm) was roughly 1.83.

**Figure 5:**
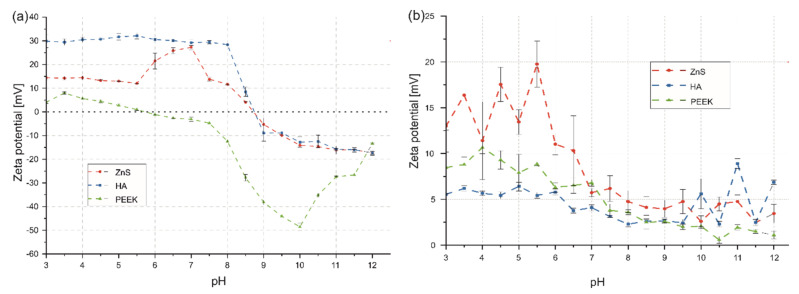
ZDP of n-HA, ZnS and PEEK particles in pure EtOH (**a**) and EtOH with the addition of 5 vol % CHp (**b**) in accordance with the suspension pH.

It was found that the ZDP of particles in EtOH depended on their type. According to that, the ZDP of PEEK particles in EtOH exhibited positive values for mixtures with a pH from 3.0 to 5.5 and negative values for suspensions with a pH above that value. ZnS and HA particles displayed similar, negative ZDP values for suspensions with pH above 8.5. Both particle types had positive values in mixtures with a pH that varied from 3.0 to 8.5 (Figure 5a). After the addition of CHp into the suspension, all coating components established positive ZDP values in the investigated pH range of 3.0–12.0, as shown in Figure 5b. Interestingly, CHp addition lowered the ZDP of ZnS particles in the mixtures with the pH range of 6.0–8.5 and changed its highest value of about 30 mV from pH = 7.0 in the pure EtOH to about 20 mV in the suspension of pH = 5.5. As for n-HA particles, the ZDP was lowered for suspensions in the pH range of 3.0–8.5, but it was increased in the range of 9.0–12.0. It reached the highest value of about 9 mV in the suspension of pH equal to 11.0. The ZDP increased significantly for PEEK particles in the suspension with CHp over the entire pH range, reaching the highest value of about 11 mV for the pH of 4. The addition of CHp contributed to the stabilization of the suspension used for coating deposition (pH = 5.36). In addition, it enabled effective cathodic co-deposition of all coating components. A similar phenomenon was observed by Pang and Zhitomirsky [49] for HA–Ag–chitosan nanocomposite coatings and in our previous study [20] for the co-deposition of PEEK, HA and MoS_2_ particles on titanium alloy substrates.

Fiołek et al. [50] also showed that the addition of chitosan polyelectrolyte allowed for the co-deposition of different polymer particles, such as PEEK and PTFE, by changing their charge from negative to positive and via the steric stabilization of the suspension. The mechanism of interaction, as a result of which PEEK acquires a positive charge in the presence of chitosan, has been studied and described by Luo and Zhitomirsky [51]. According to them, electrochemical decomposition of water takes place at the cathode, which results in a local increase in the pH value of the suspension. Neutralization of the chitosan amino group charge and adsorption of protonated chitosan to PEEK particles induces cathodic deposition. Shi et al. [52] showed that positively charged chitosan particles attracted HA nanoparticles in the suspension, resulting in electrostatic stabilization. However, Molaei et al. [53] found that the interaction between chitosan and the halloysite nanotube (HNT) causes the surface modification of HNT particles and the repulsive forces between chitosan chains induce a mechanism of steric stabilization in the suspension. Avcu et al. [37] also proved that the presence of various particle types (e.g., hydroxyapatite, bioglass, carbon nanotubes, and graphene oxide) in the suspension and their interaction with chitosan can generate steric or electrostatic stabilization. Therefore, it can be concluded that the coating deposition mechanism in this work is similar to that described above based on steric and electrostatic stabilization.

The effect of the voltage used for the deposition of coatings on their homogeneity was easily noticeable. It was observed that the deposition of particles on the substrate below the voltage level of 30 V was negligible. After deposition at a voltage of 50 V, 60 V and 70 V, coatings were inhomogeneous and thin. The coatings had numerous closed inequalities on their surface. The number of defects increased drastically for voltages above 110 V.

**Figure 6:**
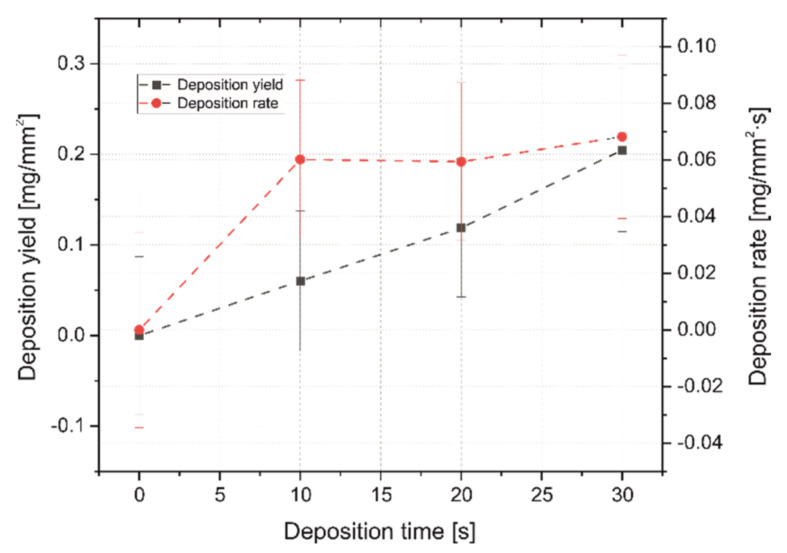
The changes in the deposition yield and the deposition rate with respect to deposition time at a constant voltage of 90 V.

The deposition yield and deposition rate of coatings was investigated (Figure 6). As EPD continued, the deposition yield grew rapidly from about 0.12 mm/mg^2^ for 20 s to 0.20 mm/mg^2^ for 30 s. The deposition rate reached its peak after 10 s and began growing dynamically, then it stabilized at 0.06 mm/mg^2^∙s until the 20th second, to finally grow slowly for the last 10 s until the end of the process, reaching a level of 0.07 mm/mg^2^∙s.

**Figure 7:**
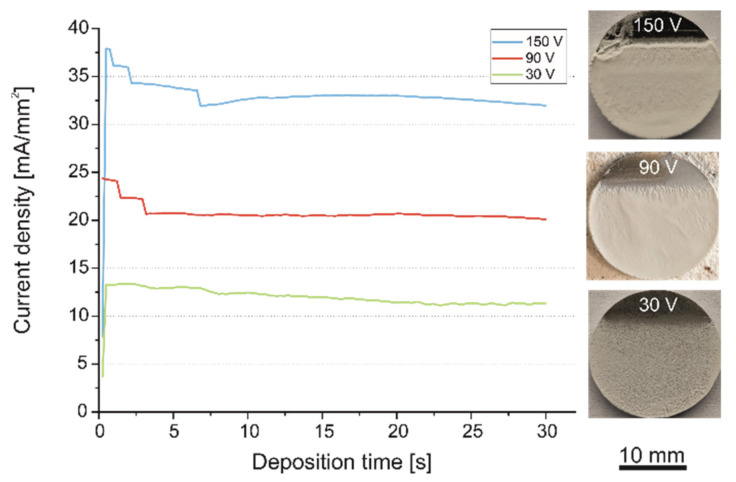
Current density variation for the EPD of n-HA/ZnS/PEEK coatings with macroscopic images of as-deposited coatings for the corresponding voltages.

Current density change and macroscopic images of coated substrates deposited at corresponding voltages of 30 V, 90 V and 150 V are shown in Figure 7.

Significant current density instability was noted for 90 V and 150 V for the measurement point of 10 s. After that, it was stable until the process was completed. Except for initial (5 s) fluctuations, the current density was constant at a level of 20 mA/mm^2^ for the voltage of 90 V. The highest current density vacillations were observed at the voltage of 150 V.

This phenomenon was manifested in coating heterogeneity. At the voltages at which greater current fluctuations occurred, larger macroscopic inhomogeneities of the obtained coatings were observed. The coatings obtained at 30 V voltage were generally homogeneous, but relatively thin. The coatings achieved at 150 V voltage were characterized by a considerable thickness and irregular surface morphology with the presence of inconsistencies and pores. Utilization of the deposition voltage of 90 V resulted in the development of coatings with a moderate amount of inconsistencies in the form of valleculae and rare pores. Comparable with our previous study on PEEK-based coatings with MoS_2_ incorporation [20], an increased concentration of HA powder in the suspension above 1 g/L multiplied the occurrence of pores and inconsistencies.

**Figure 8:**
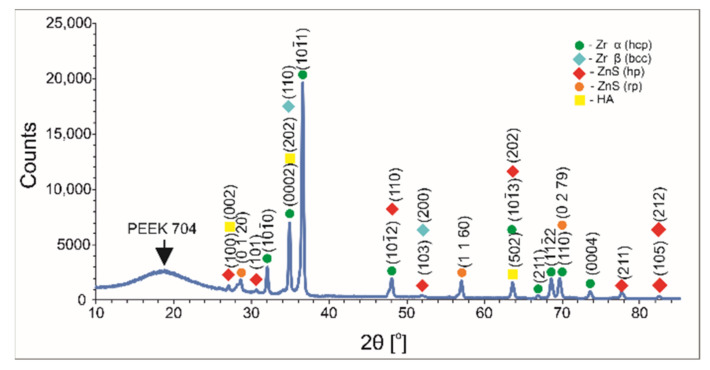
XRD patterns of n-HA/ZnS/S-PEEK coated alloy heated at 450 °C showing an amorphous PEEK structure.

Although coatings deposited at 150 V voltage were satisfactorily uniform and thick, after heat treatment they were converged, exposing a significant part of the substrate surface due to the uneven thickness and the disclosure of hidden defects under its surface. Therefore, coatings obtained at 90 V for 30 s were chosen for heat treatment and a further investigation of microstructure and properties. The determination of heating temperature was executed by an experimental approach as well as an XRD and FTIR structural analysis of the coatings. In our previous study on MoS_2_/PEEK coatings, a lower temperature of 390 °C was sufficient for PEEK sulfonation [41]. However, a different type of polymer (PEEK 708) was used and the MoS_2_ were in the form of nanosheets. It was also found that a temperature of 390 °C was not sufficient to induce amorphization and sulfonation of PEEK 704 in multicomponent HA/MoS_2_/PEEK coatings [20]. Based on these previous experiences [20,41], the heating temperature of 450 °C was selected for heating the coated substrates to ensure the occurrence of PEEK sulfonation. This was conducted for 40 min and cooled with a furnace. The coatings after the treatment were macroscopically homogeneous and dense, but single and open pores on their surface were observed. The XRD pattern of the coated alloy is presented in Figure 8. The pattern confirmed the presence of all components in the coating and exhibited an amorphous PEEK structure, indicating that the sulfonation process took place.

**Figure 9:**
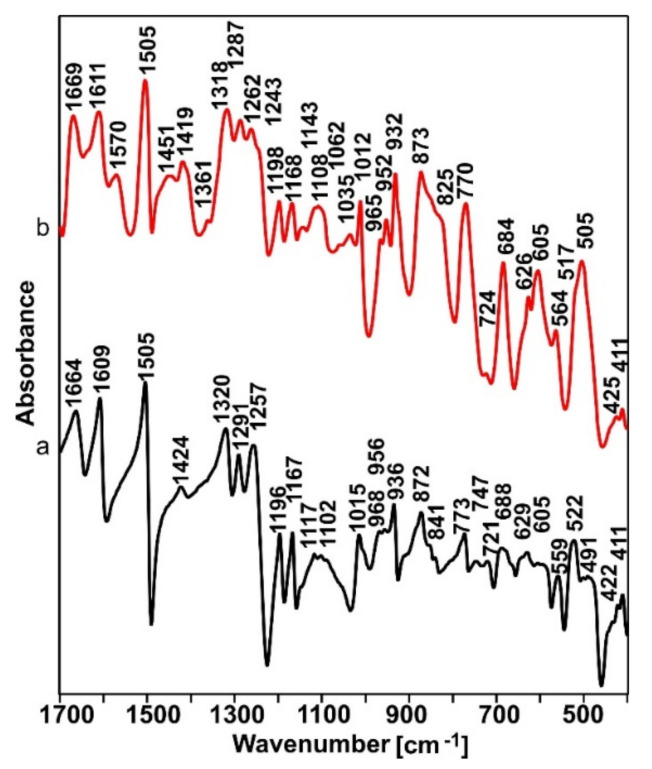
MIR spectra of PEEK powder (**a**) and the n-HA/ZnS/S-PEEK coating heated at 450 °C (**b**).

An FTIR analysis was performed to confirm the occurrence of the sulfonation process. Figure 9 shows the MIR spectra of PEEK powder (a) and the n-HA/ZnS/S-PEEK coating heated at 450 °C (b).

The first noticeable difference was the much larger half-width of most of the bands on the spectra of the PEEK coating with ZnS and HA heated at 450 °C (b). This clearly indicates a higher degree of disorder (amorphization) of the coating compared to the initial PEEK (a). Literature data indicate that the process of PEEK sulfonation leads to the amorphization of its structure [54], therefore it can be assumed that the sulfonation process took place in the coatings. In order to unequivocally confirm the occurrence of the sulfonation process, the MIR spectra were analyzed for the presence of bands characteristic for S-O and S=O bonds. This analysis is difficult due to the small quantity of sulfur ions in the investigated layers and the overlapping of the characteristic bands of S-O and S=O bonding with the characteristic bands of PEEK. Nevertheless, an in-depth analysis revealed the characteristic bands of vibrations of sulfur-oxygen bonding at approx. 1240 cm^−1^ (asymmetric O=S=O stretching) and 1035 cm^−1^ (symmetric O=S=O stretching) in spectrum (b), which did not appear in spectrum (a) [55,56]. The increase in the intensity of the bands at 684, 1035, 1112 and 1243 cm^−1^ associated with vibrations of the S-O bonds is also an observed characteristic [57].

**Figure 10:**
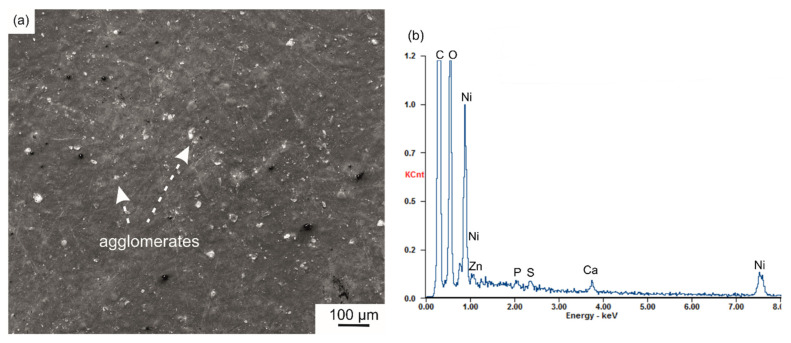
Surface morphology (**a**) and SEM-EDS spectrum (**b**) of the n-HA/ZnS/S-PEEK coating, SEM. Ni in the spectrum was generated from the sputtered conductive layer.

It was validated by microstructure observation that no spherulites were observed on the surface of coatings with S-PEEK. The PEEK changed during heat treatment from globular particles to a homogeneous, stiff and dense coating matrix (Figure 10a). The SEM-EDS microanalysis confirmed the presence of elements belonging to all coating components (Figure 10b). The coating thickness measured by contact profilometry was in the range of 55–60 μm.

**Figure 11:**
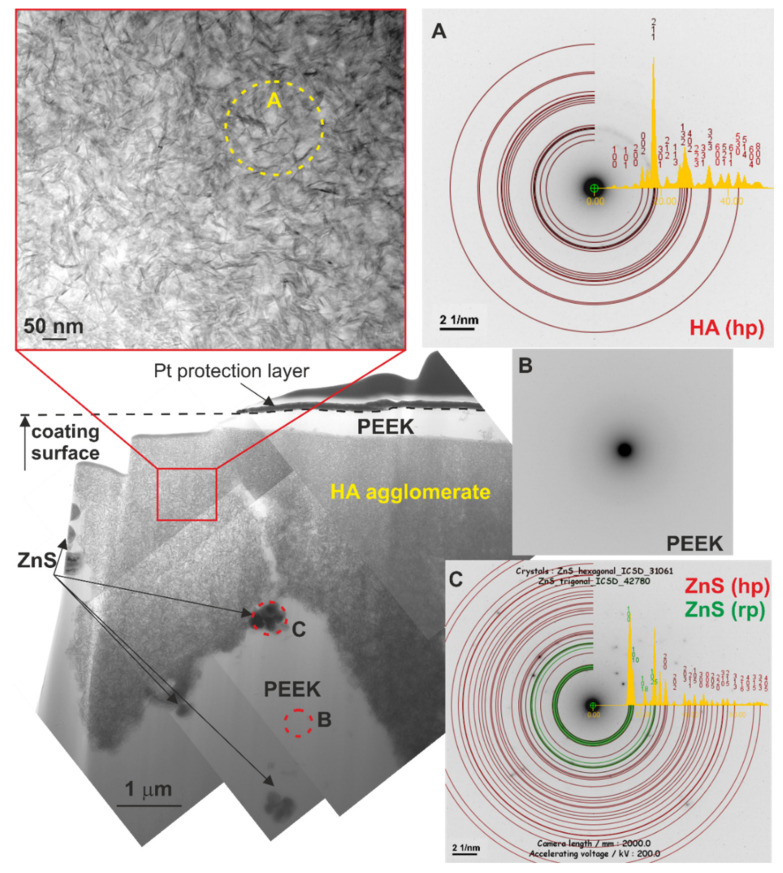
Microstructure of the n-HA/ZnS/S-PEEK coating on the cross-section and diffraction patterns (**A**–**C**) from the traverse sections of the area indicated in the figure and their interpretation, TEM.

**Figure 12:**
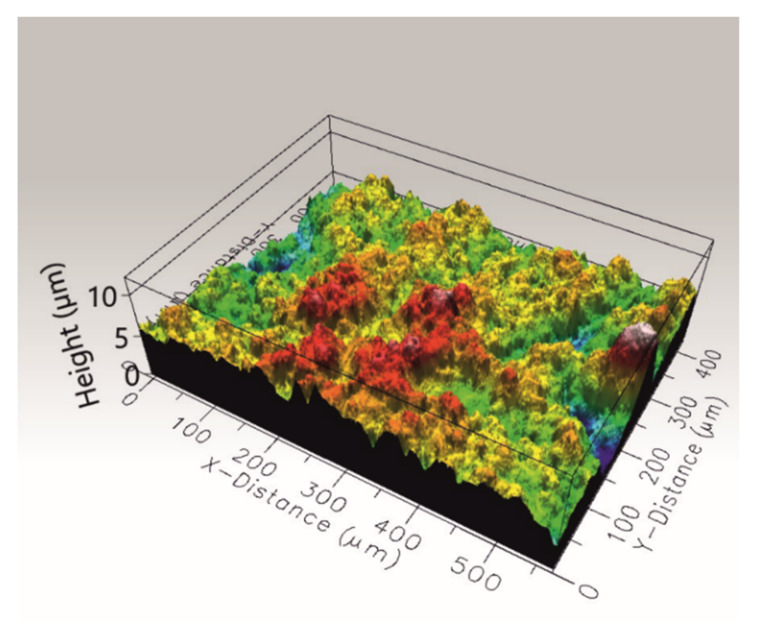
3D image of the n-HA/ZnS/PEEK coating surface after heat treatment at 450 °C, optical profilometry.

The microstructure on the traverse section of the coating’s outer region was characterized using TEM. Large agglomerates of HA nanocrystals with a diameter of about 6 μm and smaller agglomerates of ZnS particles with a diameter of about 0.5 μm were found in the amorphous polymeric matrix (Figure 11). The coating was compact in the investigated area, no closed porosity was observed and the ceramic particles were well embedded in the polymer. The n-HA/ZnS/S-PEEK coatings were characterized by a relatively high surface roughness. The arithmetic mean height Sa and root mean square height Sq parameters obtained from three averaged measurements from various locations of the coating in accordance with ISO 25178 equaled 1.27 ± 0.12 μm and 1.73 ± 0.21 μm, respectively. A characteristic image of the coating surface is shown in Figure 12.

The surface properties of the wettability angle (WA) and interfacial free energy (IFE) were examined for coatings in relation to the uncoated Zr-2.5Nb alloy and PEEK coatings without any additives. The WA and IFE for water and diiodomethane are presented in Table 1. All investigated surfaces had a WA of below 90°, while the lowest WA with water was measured for the base alloy (53 ± 8°). The coatings had a WA of slightly above 70°, which indicates mild hydrophilicity of their surfaces. These WA values were close to those obtained for pure PEEK coatings with a semicrystalline structure, which demonstrates that thermal sulfonation has no significant effect on PEEK wettability. In addition, the presence of the ZnS or n-HA particles in the coating did not change the wettability of the PEEK as they were completely embedded in the polymer. The n-HA/ZnS/S-PEEK coating revealed a considerable IFE at above 40 mN/m. However, the base alloy and pure PEEK coating exhibited higher IFE values. It is known that bare PEEK is often qualified as a material with WA values between hydrophobic and hydrophilic levels, usually in the range of 70–90° [58,59]. A WA close to the most preferable hydrophilic range of 40–70° and substantial IFE are important in the case of the osteointegration of the biomaterials with surrounding bone tissues [60,61]. Although the wettability of coatings obtained in this work was lower than for the HA/PEEK coatings developed by Bastan et al. [18], the WA of coatings was not significantly reduced compared to the PEEK coating. However, the presence of bioactive HA particles and sulfur in PEEK allows the bioactive properties of the coating to develop.

The modulus of elasticity (E_IT_) and hardness (H_IT_), examined by the method of instrumented indentation, were carried out for different loads (P_max_) of 100, 200, 400 and 1000 mN. However, only measurements obtained for 100 mN were considered because other loads exceeded the recommended penetration depth of 0.1 for the coating thickness, which increased the hardness and modulus of elasticity caused by the interaction with the alloy substrate. Relatively high hardness was obtained for the coating of 0.32 ± 0.08 GPa and elastic modulus of 4.3 ± 0.8 GPa. The hardness value for coatings was close to the hardness of 0.33 GPa obtained by Wang et al. [62] for HA/PEEK composites with 15 vol % HA produced by injection molding. Similar to our previous study on HA/MoS_2_/PEEK coatings with a semi-crystalline PEEK structure [20], the HA filler increased hardness (about 0.32 ± 0.02 GPa) compared to the unfilled PEEK 704 coating, for which H_IT_ was equal to 0.26 ± 0.03 GPa. The modulus of elasticity E_IT_ of both coatings was comparable.

**Figure 13:**
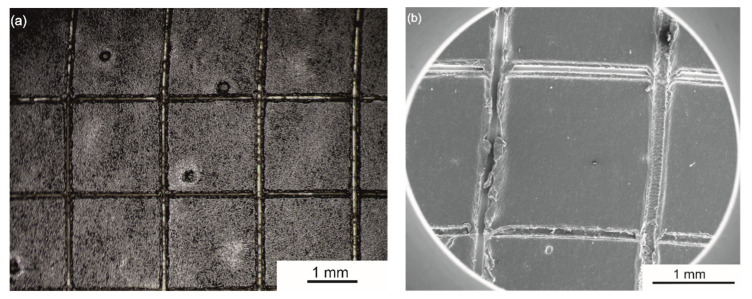
The surface of the n-HA/ZnS/S-PEEK coating (**a**) on the Zr-2.5Nb alloy after tape tests observed with a stereoscopic microscope. The enlarged detail (SEM image) of the cuts is presented in (**b**).

The adhesion strength and resistance against mechanical scratching of the coatings was assessed based on tape tests and scratch resistance tests, respectively. The n-HA/ZnS/S-PEEK coating exhibited excellent adhesion to the zirconium alloy substrate, of the highest class 5B according to ASTM D3359-17, as shown by the tape test. The coating surface after the test is shown in Figure 13. Detailed observations of the coating surface by SEM revealed minor exfoliation on a few cutting edges only.

A micro-scratch test was conducted for an accurate analysis of the scratch damage mechanism and analysis of the coating adhesion. The scratch test allows the data concerning adhesion of the coatings to the substrate to be completed quantitatively. For this purpose, the critical load at which the characteristic form of the coating failure occurred was determined. The critical load L_c1_ is defined as the load at which the first cohesive crack appears in the scratch track, L_c2_ is the load followed by the adhesive failure of the coating and a slight exposure of the substrate, and L_c3_ is the load at which the coating is completely removed from the substrate on a large area due to delamination. The relative motion of the stylus with the ascending load is the cause of the increase in the interfacial shear stress occurring between the moving and deformed surface of the coating. Furthermore, it causes the tensile stresses following it, and the pile-ups of the coating material on the edges of the scratch.

**Figure 14:**
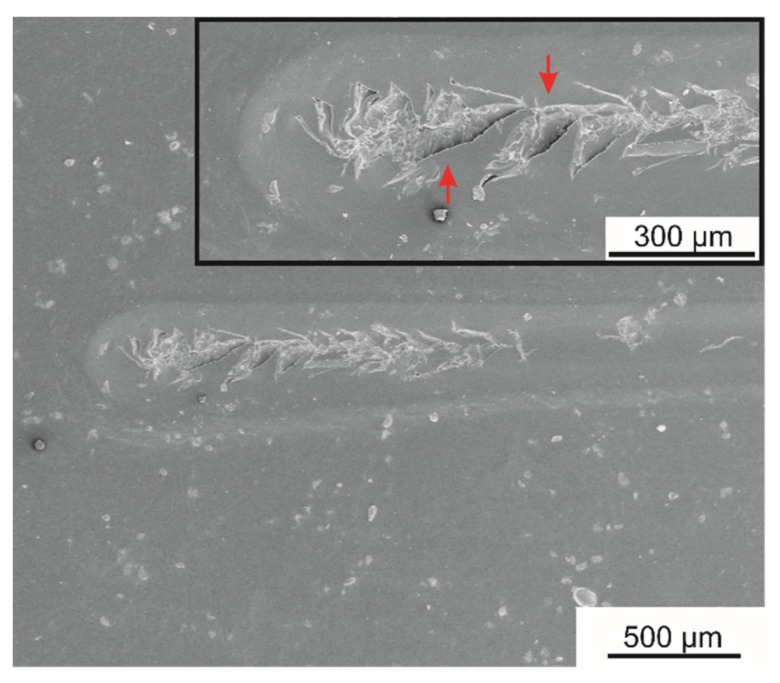
Scratch tracks in the n-HA/ZnS/S-PEEK coating in the places where the characteristic forms of failure occurred; cohesive cracks were marked with arrows.

During the tests, the acoustic emission signal was recorded, which proved their fragile nature of destruction during scratching. Cohesive cracks were observed under the load of L_c1_ = 16 N (Figure 14). The coatings deteriorated with an increase in the load and, under an L_c2_ equal to 27 N, an exposition of the substrate occurred.

**Figure 15:**
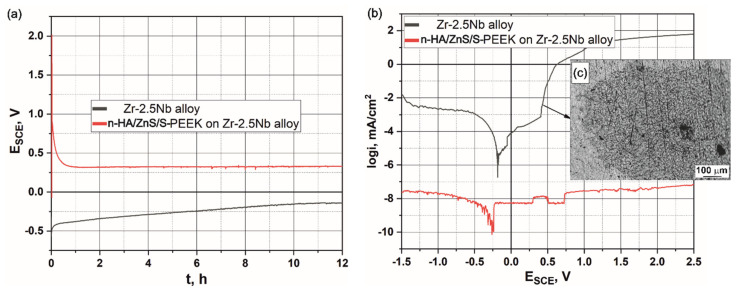
Electrochemical results for the Zr-2.5Nb alloy and the alloy coated with the n-HA/ZnS/S-PEEK coating in Ringer’s solution at 37 °C, (**a**) OCP vs. time, (**b**) Anodic polarization curves at scan rate 1 mV/s and (**c**) light microscope image of the alloy surface after the LSV experiment.

The electrochemical behavior of the base and coated alloy was investigated in Ringer’s solution at a temperature of 37 °C. For each of the samples, open circuit potentials (E_ocp_) were registered as a function of time. It was observed that the free corrosion potential (Figure 15a) slightly increased from −0.5 V for the Zr-2.5Nb alloy and reached a stable value after about 10 h of immersion in Ringer’s solution at a value of around −0.15 V. The E_ocp_ for the coated alloy shows a stable and significantly higher value of about 0.33 V. E_ocp_ values for the coated alloy indicate more noble behavior, confirming the positive effect of the n-HA/ZnS/S-PEEK coating on the substrate corrosion resistance.

The anodic polarization curves registered for the alloy and coated alloy substrate in deaerated Ringer’s solution are shown in Figure 15b. A large, weakly potential-dependent current region, which is an indication of the passive state of the coated surfaces, was observed. By contrast, the base alloy showed a continuous increase in current with potential. This is not a consequence of an active corrosion process but evidence of the continuous growth of the oxide during anodic polarization [5,63]. From about 0.4 V, a breakdown of the passive layer was observed and active dissolution of the alloy started (transpassive region). The surface morphology of the pure alloy after polarization measurements is shown in Figure 15c. In general, randomly distributed pitting spots were present on the sample surface, which confirmed the pits’ occurrence. In addition, the growth and stability of the oxide films on zirconium alloy were greatly affected by the electrolytic medium. Their corrosion properties were deteriorated by the presence of chloride ions [64,65]. The passive films were easily attacked due to the Cl^−^ migration towards and into the oxide films, which caused pitting. Thus, the decrease in the current density during the passive state (by about 4 orders of magnitude) at the anodic branch of the polarization curve indicates an improvement in their corrosion behavior. All OCP and potentiodynamic polarization data are given in Table 2.

**Figure 16:**
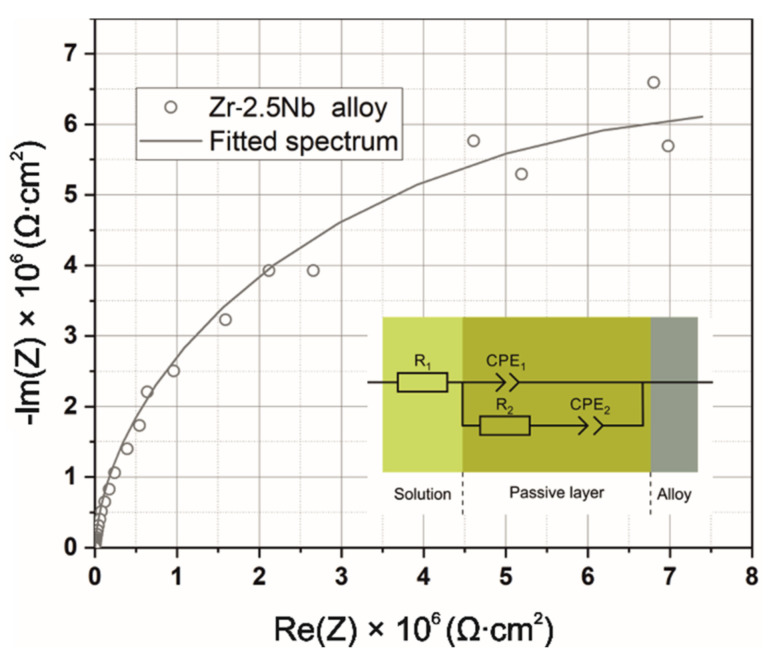
Nyquist impedance plot and equivalent circuit for the Zr-2.5Nb alloy.

In the present paper, an equivalent circuit given by the scheme [R1(Q1[R2Q2])] (shown in Figure 16) was employed to fit the EIS data for the case of a single passive film formed on the metal surface. The R1 and R2 parameters represented electrolyte resistance and the charge transfer resistance for the passive film/solution interface, respectively. Furthermore, Q1 was a constant phase element (CPE), which took into account the non-ideal capacitive behavior of the film. Circuit element Q2 described the CPE for the passive film/substrate interface. The values of the fitted parameters (R1, Q1, n1, R2, Q2, n2) are listed in Table 3. A good agreement between the experimental data and fitted curve was obtained with the χ2 of about 8 × 10^−3^. Parameters of equivalent circuit elements found by fitting for the uncoated Zr-2.5Nb substrate indicate that, due to a rather high value of exponent n1 ≈ 0.975 for the constant phase element CPE1, its nature is capacitive, whereas in the case of the constant phase element CPE2, a more resistive behavior dominates, n2 ≈ 0.174. On the other hand, it was impossible to register impedance spectra for the coated samples due to extremely low current values (please refer to those obtained in the LSV experiment for the coated alloy), resulting in very high impedance—all registered spectra resembled clouds of randomly scattered points and are therefore not presented in this paper.

**Figure 17:**
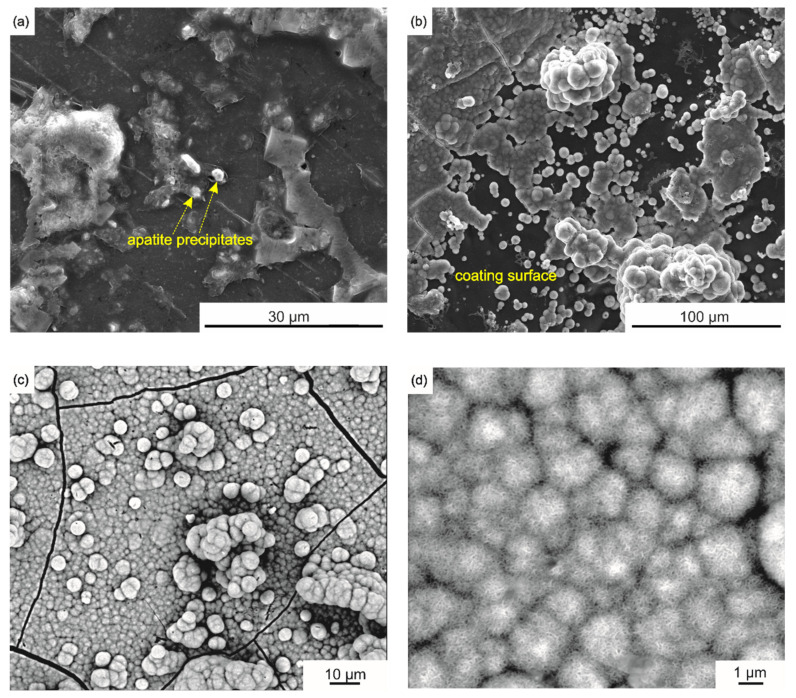
SEM images of the coated zirconium alloy after incubation in the SBF for 7 days (**a**), 10 days (**b**) and 21 days (**c**,**d**). (**c**) shows cauliflower-like morphology, while (**d**) shows flake-like morphology of the apatite layer.

The initial response of the n-HA/ZnS/S-PEEK coated alloy after its incubation in SBF was studied using the modified Kokubo test [45]. After soaking in SBF for 1 and 3 days, no mass gain of samples was detected and no HA formation was recognized on the coating surface. This behaviour may be due to the specific microstructure of the coatings. The n-HA separate nanoparticles and their agglomerates were completely embedded in the polymer matrix (Figure 11), which significantly delayed the release of Ca and P ions. Thus the formation of carbonated hydroxyapatite on the surface of the coating was difficult. A longer incubation time (7 and 10 days) resulted in slight changes (about 1 mg for 7 days and 2 mg after 10 days) in the weight of the sample, thereby indicating that apatite had started to nucleate and grow, which is visible in Figure 17a,b. After incubation for 21 days, the mass gain of the apatite layer was 8 mg, and its thickness, determined from the differences in the sample mass before and after the test, taking into account the HA density of 3.16 g/cm^3^, was about 10 μm. The SBF was changed weekly during the test. The pH of the initial SBF and that measured at RT after 1, 2 and 3 weeks of the test was the same at a level of 7.95 ± 0.02. The pH values depended on the SBF temperature and decreased to about 7.40 at 36.5 °C. The pH of SBF measured at the test temperature (36.5 °C) increased after 1 day to about 7.59 and after 7 days of immersion to 7.61. After 10, 14 and 21 days of the immersion pH equalled 7.66. No further increase in the pH of the SBF was observed. This may be due to the weekly change of the solution. Similarly, Khazeni et al. [66] showed that the pH of SBF increased over time to a level above 7.60. However, their study was conducted for 120 h of immersion of HA-CNTs composite coatings on magnesium alloy at a temperature of 37 ± 1 °C, without changing SBF.

It was found that the first small precipitates of apatite appeared after 7 days (Figure 17a). Precipitates grew rapidly, which became visible on the coating surface after 10 days of the test (Figure 17b). A characteristic cauliflower-like apatite morphology was observed on the whole coating surface after a three-week-long test (Figure 17c). These changes on the sample surfaces correspond well with the changes in pH and weight of the samples described above. A similar layer with cauliflower-like morphology was obtained on the HA/PEEK coating after a bioactivity test in SBF by Bastan et al. [18]. Further SEM observation revealed that the whole coating surface was covered by apatite precipitates with flake morphology arranged in spherical aggregates with a size of 1–3 µm (Figure 17d). The presence of apatite on the n-HA/ZnS/S-PEEK coating indicates the high potential bioactivity of the coatings. The SEM-EDS microanalysis performed for a large coating area of 1100 μm × 930 μm revealed that the Ca:P atomic fraction was roughly 1.6. Such a value corresponds to non-stoichiometric carbonate hydroxyapatite.

**Figure 18:**
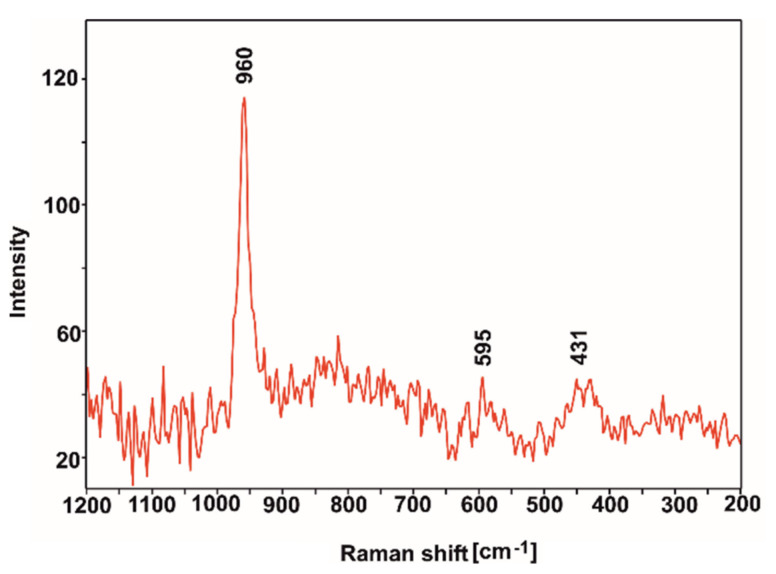
Raman spectrum of the layer formed after incubation in SBF.

In order to identify the phases that formed on the surface of the tested sample after the incubation process in SBF, Raman microscopy studies were carried out, focusing the beam on the characteristic layer visible in the SEM images (Figure 17c,d). As is clearly noticeable, the obtained Raman spectrum (Figure 18) shows an intense band at 960 cm^−1^ and two low-intensity bands at 595 and 431 cm^−1^. Such an arrangement of bands [56,67] and the results of the EDS microanalysis allow us to assume that hydroxyapatite is present on the surface of the tested sample after incubation.

Carbonate hydroxyapatite is the most common apatite occurring in human bone [68]. It is the preferable form of stoichiometric HA with low crystallinity and improved solubility, which leads to full resorbility and more accessible bone formation [69]. Hence, its presence may indicate the potential of bioactivity and osteoconductivity of the obtained coating.

## 4. Conclusions

In this study, the possibility of the co-deposition of PEEK 704 with ZnS and HA nanoparticles for obtaining n-HA/ZnS/S-PEEK coatings was discussed. The main conclusions are:

1. Duplex treatment based on EPD and heat treatment allowed for the development of homogeneous multicomponent coatings on Zr-2.5Nb alloy substrates. Coatings were obtained at a constant voltage of 90 V during 30 s. The n-HA/ZnS/PEEK coating deposition rate and yield was the highest at 30 s of the process.
2. Heat treatment caused PEEK transformation from particles into a continuous phase, in which ceramic particles were embedded. As a result of heat treatment at a temperature of 450 °C, a sulfonation process occurred in the coating, leading to the formation of an amorphous PEEK matrix. The surface of n-HA/ZnS/S-PEEK coatings showed high roughness.
3. The HA/ZnS/S-PEEK coatings demonstrated excellent adhesion to the alloy substrate and moderate scratch resistance.
4. Coatings with the addition of HA displayed higher hardness and modulus of elasticity compared to the PEEK coating without additives.
5. LSV and EIS experiment results indicate a significant improvement in corrosion resistance of the coated Zr-2.5Nb alloy compared to the bare substrate. However, due to the very low current densities registered for this coating, it was not possible to obtain any meaningful EIS data—the observed impedance values were above the limits of the potentiostat/galvanostat used, which in turn suggests a very high stability of the n-HA/ZnS/S-PEEK coating in aggressive Ringer’s solution.
6. After immersion in simulated body fluid, an apatite-like layer was formed on the n-HA/ZnS/S-PEEK coating surface.

## Figures and Tables

**Table 1:** Water and diiodomethane wettability angle and interfacial free energy of the Zr-2.5Nb alloy, PEEK and n-HA/ZnS/S-PEEK coatings.

Material	WA [°]		IFE [mN/m]	
	H_2_O	CH_2_I_2_	Polar	Disperse
Zr-2.5Nb alloy	53.0 ± 8.0	48.3 ± 2.0	53.6 ± 6.0	
			18.4 ± 4.9	35.2 ± 1.1
PEEK coating	71.1 ± 9.0	25.8 ± 1.4	51.3 ± 4.1	
			5.4 ± 3.6	45.9 ± 0.5
n-HA/ZnS/S-PEEK coating	73.1 ± 3.5	36.9 ± 3.4	46.8 ± 3.1	
			5.7 ± 1.5	41.2 ± 1.6

**Table 2:** Corrosion parameters for Zr-2.5Nb and coated alloy obtained from the analysis of OCP and polarization results.

Samples	OCP [V]	E_K-A_ [V]	Icorr [µA/cm^2^]
Zr-2.5Nb	−0.15	−0.18	93
n-HA/ZnS/S-PEEK coated Zr-2.5Nb	0.33	−0.26	0.00002

**Table 3:** Equivalent circuit parameters calculated by means of fitting for the Zr-2.5Nb alloy.

Element of Equivalent Circuit	Quantity	Value
R1	Rs (solution resistance) (Ω)	36.72
CPE1	Y0 (S)	4.918 × 10^−6^
	n1	0.978
R2	Rp (polarization resistance) (Ω)	2248.7
CPE2	Y0 (S)	1.850 × 10^−7^
	n2	0.170
	χ^2^	8 × 10^−3^

## Data Availability

The data presented in this study are available on request from the corresponding author.

## References

[1] Vitti R.P., Catelan A., Amaral M., Pacheco R.R. (2019). Zirconium in Dentistry.

[2] Chopra D., Gulati K., Ivanovski S. (2021). Micro + Nano: Conserving the Gold Standard Microroughness to Nanoengineer Zirconium Dental Implants. ACS Biomater. Sci. Eng..

[3] Matuła I., Dercz G., Barczyk J. (2020). Titanium/Zirconium functionally graded materials with porosity gradients for potential biomedical applications. Mater. Sci. Technol..

[4] Branzoi I.V., Iordoc M., Codescu M. (2008). Electrochemical studies on the stability and corrosion resistance of new zirconium-based alloys for biomedical applications. Surf. Interface Anal..

[5] Zhou F., Wang B., Qiu K., Lin W., Li L., Wang Y., Nie F., Zheng Y. (2012). Microstructure, corrosion behavior and cytotoxicity of Zr–Nb alloys for biomedical application. Mater. Sci. Eng. C.

[6] Zhou F., Qiu K., Bian D., Zheng Y., Lin J. (2014). A Comparative in vitro Study on Biomedical Zr–2.5X (X = Nb, Sn) Alloys. J. Mater. Sci. Technol..

[7] Mehjabeen A., Song T., Xu W., Tang H.P., Qian M. (2018). Zirconium Alloys for Orthopaedic and Dental Applications. Adv. Eng. Mater..

[8] Corni I., Neumann N., Eifler D., Boccaccini A.R. (2008). Polyetheretherketone (PEEK) Coatings on Stainless Steel by Electrophoretic Deposition. Adv. Eng. Mater..

[9] Boccaccini A.R., Peters C.T., Roether J.A., Eifler D., Misra S.K., Minay E.J. (2006). Electrophoretic deposition of polyetheretherketone (PEEK) and PEEK/Bioglass^®^ coatings on NiTi shape memory alloy wires. J. Mater. Sci..

[10] Panayotov I.V., Orti V., Cuisinier F., Yachouh J. (2016). Polyetheretherketone (PEEK) for medical applications. J. Mater. Sci. Mater. Med..

[11] Abdulkareem M.H., Abdalsalam A.H., Bohan A.J. (2019). Influence of chitosan on the antibacterial activity of composite coating (PEEK /HAp) fabricated by electrophoretic deposition. Prog. Org. Coatings.

[12] Kurtz S.M. (2019). An Overview of PEEK Biomaterials.

[13] Heary R.F., Parvathreddy N., Sampath S., Agarwal N. (2017). Elastic modulus in the selection of interbody implants. J. Spine Surg..

[14] Rehman M.A.U., Bastan F.E., Haider B., Boccaccini A.R. (2017). Electrophoretic deposition of PEEK/bioactive glass composite coatings for orthopedic implants: A design of experiments (DoE) study. Mater. Des..

[15] Rehman M.A.U., Bastan F.E., Nawaz Q., Goldmann W.H., Maqbool M., Virtanen S., Boccaccini A.R. (2018). Electrophoretic deposition of lawsone loaded bioactive glass (BG)/chitosan composite on polyetheretherketone (PEEK)/BG layers as antibacterial and bioactive coating. J. Biomed. Mater. Res. Part A.

[16] Virk R.S., Rehman M.A.U., Munawar M.A., Schubert D.W., Goldmann W.H., Dusza J., Boccaccini A.R. (2019). Curcumin-Containing Orthopedic Implant Coatings Deposited on Poly-Ether-Ether-Ketone/Bioactive Glass/Hexagonal Boron Nitride Layers by Electrophoretic Deposition. Coatings.

[17] Moskalewicz T., Zych A., Łukaszczyk A., Cholewa-Kowalska K., Kruk A., Dubiel B., Radziszewska A., Berent K., Gajewska M. (2017). Electrophoretic Deposition, Microstructure, and Corrosion Resistance of Porous Sol–Gel Glass/Polyetheretherketone Coatings on the Ti-13Nb-13Zr Alloy. Met. Mater. Trans. A.

[18] Baştan F.E., Rehman M.A.U., Avcu Y.Y., Avcu E., Üstel F., Boccaccini A.R. (2018). Electrophoretic co-deposition of PEEK-hydroxyapatite composite coatings for biomedical applications. Colloids Surf. B Biointerfaces.

[19] Fiołek A., Zimowski S., Kopia A., Moskalewicz T. (2019). The Influence of Electrophoretic Deposition Parameters and Heat Treatment on the Microstructure and Tribological Properties of Nanocomposite Si3N4/PEEK 708 Coatings on Titanium Alloy. Coatings.

[20] Kuśmierczyk F., Zimowski S., Łukaszczyk A., Kopia A., Cieniek L., Moskalewicz T. (2021). Development of Microstructure and Properties of Multicomponent MoS2/HA/PEEK Coatings on a Titanium Alloy Via Electrophoretic Deposition and Heat Treatment. Met. Mater. Trans. A.

[21] Seuss S., Heinloth M., Boccaccini A.R. (2016). Development of bioactive composite coatings based on combination of PEEK, bioactive glass and Ag nanoparticles with antibacterial properties. Surf. Coatings Technol..

[22] Rehman M.A.U., Bastan F.E., Nawaz A., Nawaz Q., Wadood A. (2020). Electrophoretic deposition of PEEK/bioactive glass composite coatings on stainless steel for orthopedic applications: An optimization for in vitro bioactivity and adhesion strength. Int. J. Adv. Manuf. Technol..

[23] Ma R., Tang T. (2014). Current Strategies to Improve the Bioactivity of PEEK. Int. J. Mol. Sci..

[24] Bordea I.R., Candrea S., Alexescu G.T., Bran S., Băciuț M., Băciuț G., Lucaciu O., Dinu C.M., Todea D.A. (2020). Nano-hydroxyapatite use in dentistry: A systematic review. Drug Metab. Rev..

[25] Sanpo N., Tan M.L., Cheang P., Khor K. (2008). Antibacterial Property of Cold-Sprayed HA-Ag/PEEK Coating. J. Therm. Spray Technol..

[26] Wang J., Gong X., Hai J., Li T. (2018). Synthesis of silver–hydroxyapatite composite with improved antibacterial properties. Vacuum.

[27] Akhtar M.A., Ilyas K., Dlouhý I., Siska F., Boccaccini A.R. (2020). Electrophoretic Deposition of Copper(II)–Chitosan Complexes for Antibacterial Coatings. Int. J. Mol. Sci..

[28] Bi Q., Song X., Chen Y., Zheng Y., Yin P., Lei T. (2020). Zn-HA/Bi-HA biphasic coatings on Titanium: Fabrication, characterization, antibacterial and biological activity. Colloids Surf. B Biointerfaces.

[29] Kumar R., Sakthivel P., Mani P. (2019). Structural, optical, electrochemical, and antibacterial features of ZnS nanoparticles: Incorporation of Sn. Appl. Phys. A.

[30] Hojamberdiev M., Piccirillo C., Cai Y., Kadirova Z., Yubuta K., Ruzimuradov O. (2019). ZnS-containing industrial waste: Antibacterial activity and effects of thermal treatment temperature and atmosphere on photocatalytic activity. J. Alloy. Compd..

[31] Aqeel M., Ikram M., Asghar A., Haider A., Ul-Hamid A., Naz M., Imran M., Ali S. (2020). Synthesis of capped Cr-doped ZnS nanoparticles with improved bactericidal and catalytic properties to treat polluted water. Appl. Nanosci..

[32] Ouyang L., Zhao Y., Jin G., Lu T., Li J., Qiao Y., Ning C., Zhang X., Chu P., Liu X. (2016). Influence of sulfur content on bone formation and antibacterial ability of sulfonated PEEK. Biomaterials.

[33] Montero J.F., Tajiri H.A., Barra G.M.D.O., Fredel M.C., Benfatti C.A., Magini R.S., Pimenta A.L., Souza J.C. (2017). Biofilm behavior on sulfonated poly(ether-ether-ketone) (sPEEK). Mater. Sci. Eng. C.

[34] Ma R., Wang J., Li C., Ma K., Wei J., Yang P., Guo D., Wang K., Wang W. (2020). Effects of different sulfonation times and post-treatment methods on the characterization and cytocompatibility of sulfonated PEEK. J. Biomater. Appl..

[35] Meng Z., Qin G., Zhang B., Bai J. (2004). DNA damaging effects of sulfur dioxide derivatives in cells from various organs of mice. Mutagenesis.

[36] Meng Z., Liu Y., Wu D. (2005). Effect of Sulfur Dioxide Inhalation on Cytokine Levels in Lungs and Serum of Mice. Inhal. Toxicol..

[37] Avcu E., Bastan F.E., Abdullah H.Z., Rehman M.A.U., Avcu Y.Y., Boccaccini A.R. (2019). Electrophoretic deposition of chitosan-based composite coatings for biomedical applications: A review. Prog. Mater. Sci..

[38] Moskalewicz T., Seuss S., Boccaccini A.R. (2013). Microstructure and properties of composite polyetheretherketone/Bioglass^®^ coatings deposited on Ti–6Al–7Nb alloy for medical applications. Appl. Surf. Sci..

[39] Boccaccini A.R., Gerhardt L.-C., Rebeling S., Blaker J.J. (2005). Fabrication, characterisation and assessment of bioactivity of poly (d,l lactid acid) (PDLLA)/TiO_2_ nanocomposite films. Compos. Part A Appl. Sci. Manuf..

[40] Zhitomirsky D., Roether J., Boccaccini A. (2009). Electrophoretic deposition of bioactive glass/polymer composite coatings with and without HA nanoparticle inclusions for biomedical applications. J. Mater. Process. Technol..

[41] Moskalewicz T., Kruk A., Sitarz M., Kopia A. (2019). Effect of the Processing and Heat Treatment Route on the Microstructure of MoS2/Polyetheretherketone Coatings Obtained by Electrophoretic Deposition. J. Electrochem. Soc..

[42] Najeeb S., Khurshid Z., Matinlinna J.P., Siddiqui F., Nassani M.Z., Baroudi K. (2015). Nanomodified Peek Dental Implants: Bioactive Composites and Surface Modification—A Review. Int. J. Dent..

[43] Brasselet C., Pierre G., Dubessay P., Dols-Lafargue M., Coulon J., Maupeu J., Vallet-Courbin A., De Baynast H., Doco T., Michaud P. (2019). Modification of Chitosan for the Generation of Functional Derivatives. Appl. Sci..

[44] Oliver W.C., Pharr G.M. (1992). An improved technique for determining hardness and elastic modulus using load and displacement sensing indentation experiments. J. Mater. Res..

[45] Tanahashi M., Yao T., Kokubo T., Minoda M., Miyamoto T., Nakamura T., Yamamuro T. (1994). Apatite Coating on Organic Polymers by a Biomimetic Process. J. Am. Ceram. Soc..

[46] Perović V., Weatherly G.C. (1989). The β to α transformation in a Zr-2.5 wt% Nb alloy. Acta Met..

[47] Srivastava D., Dey G.K., Banerjee S. (1995). Evolution of microstructure during fabrication of Zr-2.5 Wt pct Nb alloy pressure tubes. Met. Mater. Trans. A.

[48] Moskalewicz T., Zimowski S., Zych A., Łukaszczyk A., Reczyńska K., Pamuła E. (2018). Electrophoretic Deposition, Microstructure and Selected Properties of Composite Alumina/Polyetheretherketone Coatings on the Ti-13Nb-13Zr Alloy. J. Electrochem. Soc..

[49] Pang X., Zhitomirsky I. (2008). Electrodeposition of hydroxyapatite–silver–chitosan nanocomposite coatings. Surf. Coatings Technol..

[50] Fiołek A., Zimowski S., Kopia A., Sitarz M., Moskalewicz T. (2020). Effect of Low-Friction Composite Polymer Coatings Fabricated by Electrophoretic Deposition and Heat Treatment on the Ti-_6_Al-_4_V Titanium Alloy’s Tribological Properties. Met. Mater. Trans. A.

[51] Luo D., Zhitomirsky I. (2015). Electrophoretic Deposition of Polyetheretherketone Composites, Containing Huntite and Alumina Platelets. J. Electrochem. Soc..

[52] Shi Y.Y., Li M., Liu Q., Jia Z.J., Xu X.C., Cheng Y., Zheng Y.F. (2016). Electrophoretic deposition of graphene oxide reinforced chitosan–hydroxyapatite nanocomposite coatings on Ti substrate. J. Mater. Sci. Mater. Med..

[53] Molaei A., Amadeh A., Yari M., Afshar M. (2016). Structure, apatite inducing ability, and corrosion behavior of chitosan/halloysite nanotube coatings prepared by electrophoretic deposition on titanium substrate. Mater. Sci. Eng. C.

[54] Zaidi S.M.J. (2003). Polymer Sulfonation A Versatile Route to Prepare Proton-Conducting Membrane Material for Advanced Tech-nologies. Arab. J. Sci. Eng..

[55] Jin X., Bishop M.T., Ellis T.S., Karasz F.E. (1985). A sulphonated poly(aryl ether ketone). Br. Polym. J..

[56] Jastrzębski W., Sitarz M., Rokita M., Bułat K. (2011). Infrared spectroscopy of different phosphates structures. Spectrochim. Acta Part A Mol. Biomol. Spectrosc..

[57] Li Y., Liu X., Li Z., Ren Y., Wang Y., Zhang W. (2021). Preparation, characterization and application of red mud, fly ash and desulfurized gypsum based eco-friendly road base materials. J. Clean. Prod..

[58] Rupp F., Gittens R.A., Scheideler L., Marmur A., Boyan B.D., Schwartz Z., Geis-Gerstorfer J. (2014). A review on the wettability of dental implant surfaces I: Theoretical and experimental aspects. Acta Biomater..

[59] Novotna Z., Reznickova A., Rimpelova S., Vesely M., Kolska Z., Svorcik V. (2015). Tailoring of PEEK bioactivity for improved cell interaction: Plasma treatment in action. RSC Adv..

[60] Elawadly T.A., Radi I.A.W., El Khadem A., Osman R.B. (2017). Can PEEK Be an Implant Material? Evaluation of Surface Topography and Wettability of Filled Versus Unfilled PEEK with Different Surface Roughness. J. Oral Implant..

[61] Liber-Kneć A., Łagan S. (2021). Surface Testing of Dental Biomaterials—Determination of Contact Angle and Surface Free Energy. Materials.

[62] Wang L., Weng L., Song S., Zhang Z., Tian S., Ma R. (2011). Characterization of polyetheretherketone–hydroxyapatite nanocomposite materials. Mater. Sci. Eng. A.

[63] Farina S.B., Sanchez A.G., Ceré S. (2015). Effect of Surface Modification on the Corrosion Resistance of Zr-2.5Nb as Material for Permanent Implants. Procedia Mater. Sci..

[64] Satpati A.K., Phadnis S.V., Sundaresan R.I. (2005). Electrochemical and XPS studies and the potential scan rate dependent pitting corrosion behavior of Zircaloy-2 in 5% NaCl solution. Corros. Sci..

[65] Palit G.C., Gadiyar H.S. (1987). Pitting Corrosion of Zirconium in Chloride Solution. Corrosion.

[66] Khazeni D., Saremi M., Soltani R. (2019). Development of HA-CNTs composite coating on AZ31 Magnesium alloy by cathodic electrodeposition. Part 2: Electrochemical and in-vitro behavior. Ceram. Int..

[67] Stammeier J.A., Purgstaller B., Hippler D., Mavromatis V., Dietzel M. (2018). In-situ Raman spectroscopy of amorphous calcium phosphate to crystalline hydroxyapatite transformation. MethodsX.

[68] Januariyasa I.K., Ana I.D., Yusuf Y. (2020). Nanofibrous poly(vinyl alcohol)/chitosan contained carbonated hydroxyapatite nanoparticles scaffold for bone tissue engineering. Mater. Sci. Eng. C.

[69] Ezekiel I., Kasim S.R., Ismail Y.M.B., Noor A.-F.M. (2018). Nanoemulsion synthesis of carbonated hydroxyapatite nanopowders: Effect of variant CO32−/PO43− molar ratios on phase, morphology, and bioactivity. Ceram. Int..

